# Supplementary figures and images for: Poly(ADP-ribosylation) is present in murine sciatic nerve fibers and is altered in a Charcot-Marie-Tooth-1E neurodegenerative model
Source: PeerJ. 2017 May 10;5:e3318. doi: 10.7717/peerj.3318 (PMC5428328; doi:10.7717/peerj.3318)

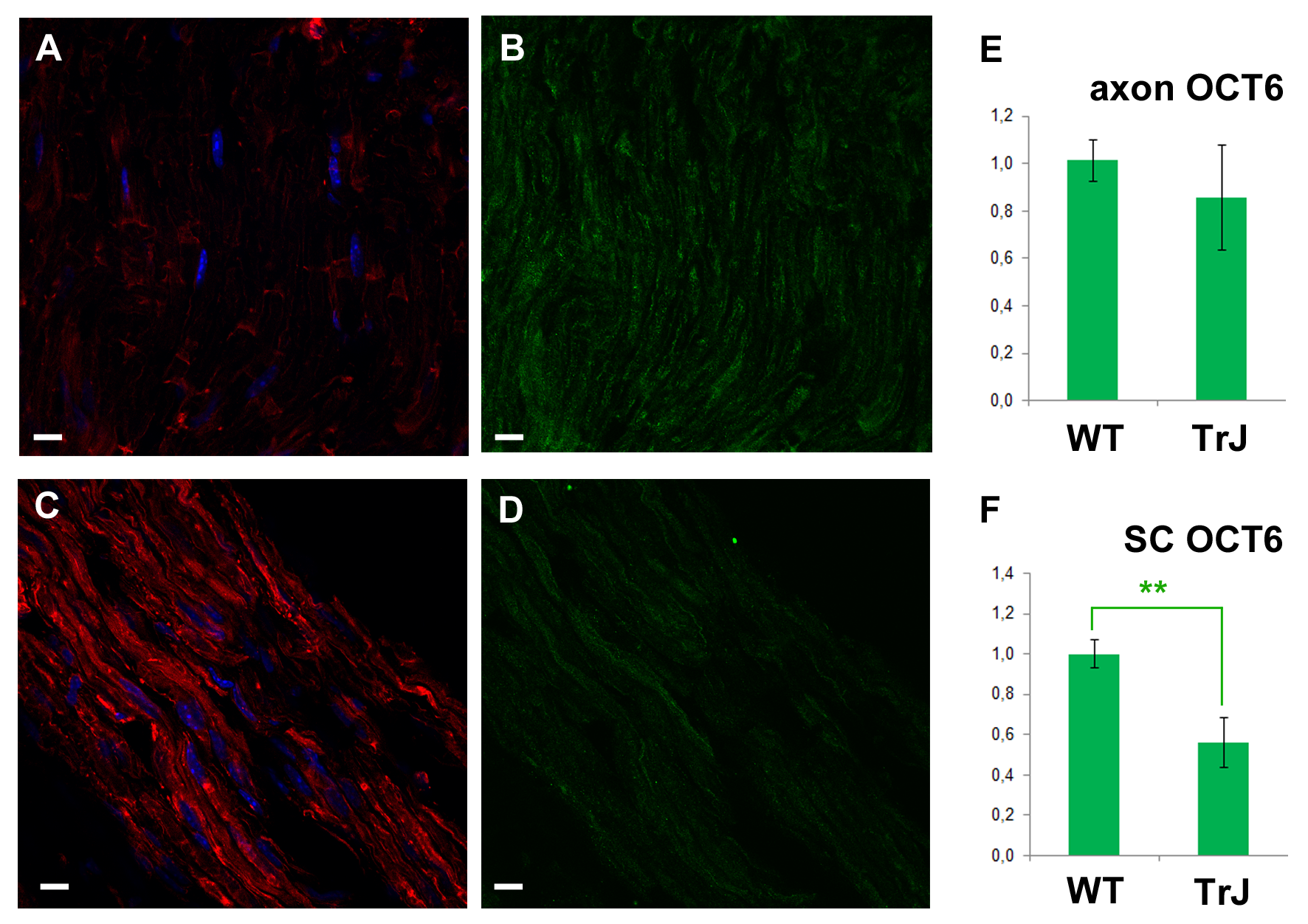

Supplement: Figure S1 — Green: OCT6, red: F-actin, blue: DAPI. (A) and (B): WT. (C) and (D): TrJ. PFA-fixed, permeabilized mice nerve cryosections or teased nerves were exposed to 0.1% sodium borohydride to block aldehydes and ketones. Nonspecific bonds were blocked with 5% goat serum in incubation buffer (IB; 150 mM glicine, 1% BSA in PHEM) 30 min at 37 °C. Then the samples were incubated 16 h at 4 °C with rabbit anti-OCT6 (ab31766; 1:100) in IB. After washing, the signal was detected with goat anti-rabbit (Invitrogen A11008; 1:1000) in IB. Counterstaining of actin and DNA was done with phalloidin-543 (Invitrogen A22283, 1:100) and DAPI (Invitrogen D1306, 1:1000). Finally, ProLong Antifade (Invitrogen) was used as mounting media. Confocal microscopy images were taken under identical conditions for WT and Tr-J in each experiment, using as a reference the control without primary antibody. OCT6 signal was measured along the fiber diameter in at least 100 DAPI-negative ROIs per genotype. The length of the ROI was normalized and divided into 10 equal parts. It was assumed that the intervals from 0 to 30% and from 70 to 100% correspond to SC whereas the 30 to 70% range corresponds to axons. Data were averaged and normalized by mean WT SC OCT6 of the corresponding experiment and expressed as mean ± s.e.m. (E): Relative intensity of OCT6 in the axons of WT and Tr-J mice. (F): Counterpart of (E) in SC cytoplasm. Bar: 10 µm. [file peerj-05-3318-s001.png]

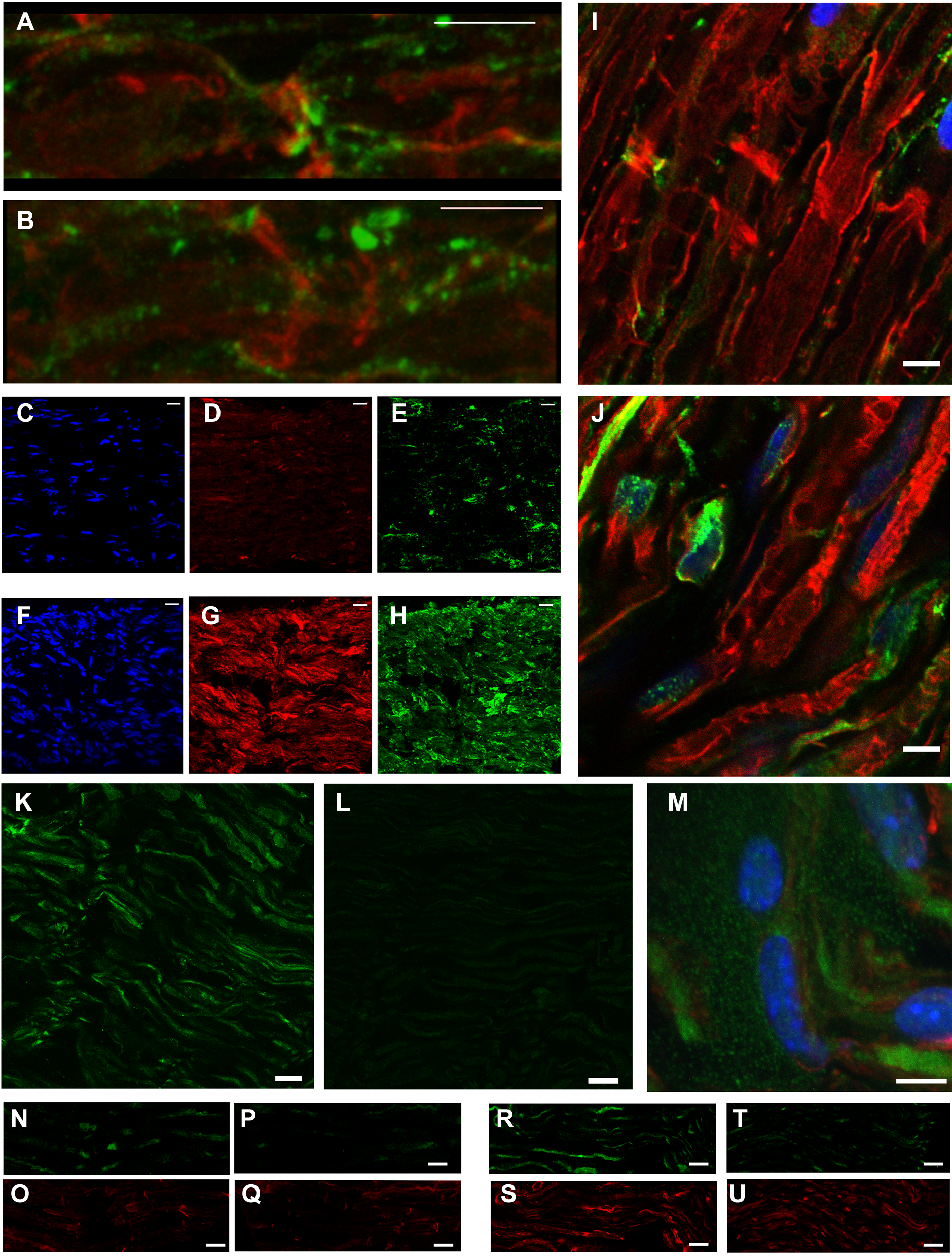

Supplement: Figure S2 — As in the main paper Figures, Green represents PAR, red: F-actin (phalloidin-546) and blue: DAPI. Top (A–J): direct immunodetection of PAR with an alternative anti-PAR antibody. ENZO mice anti-PAR antibody (BML-SA216) conjugated to Alexa Fluor488 (SIGMA MX488AS20 kit) in WT (A–E, I) and Tr-J (F–H, J) sciatic nerves. (A) and (B): WT sciatic nerve details showing (A): paranodal region and (B): Schmidt-Lanterman incisure. Merged channels 3-D reconstructions from 100x zoom 3 confocal stacks. Bar: 5 µm. (C–J): comparison of WT and Tr-J nerves. (C–E, I): WT nerve; (F–H, J); Tr-J nerve. (C–H): Overview. Single-channel z-projections of 40x stacks. Bar: 15 µm. (I and J): 100x zoom 3 single confocal slices. Bar: 5 µm. Two independent IHF experiments were carried out using ENZO mouse anti-PAR antibody, first indirectly and then directly with similar results (being the indirect IHF dirtier). Bottom (K–U): digestion of PAR with PARG. Detection with BD anti-PAR antibody. (K) and (L): Adjacent fixed cryosections from Tr-J nerves were respectively incubated in PARG reaction buffer only (50 mM KH2PO4, 50 mM KCl, 10 mM β-mercaptoethanol, 10% glycerol, 1 mM DTT and 0.1% Triton X-100) or buffer + PARG (SIGMA SRP8023; 0.5 ng/µL) during 24 h at RT. Then, the IHF was done as usual. 100x single confocal slices. Bar: 15 µm. (M): cloud of semi-digested PAR as seen in a z-projection from a 100x zoom 3 stack. Bar: 5 µm. Sample images of WT (N, P) and Tr-J (R, T), non-digested (N,R) vs digested (P,T) sciatic nerve PAR. (O–U): correspondent F-actin images. Bar: 10 µm. These are representative images from four independent digestions. [file peerj-05-3318-s002.png]
